# Synergistic tomographic image reconstruction: part 1

**DOI:** 10.1098/rsta.2020.0189

**Published:** 2021-06-28

**Authors:** Charalampos Tsoumpas, Jakob Sauer Jørgensen, Christoph Kolbitsch, Kris Thielemans

**Affiliations:** ^1^ Biomedical Imaging Science Department, University of Leeds, Leeds, West Yorkshire, UK; ^2^ Biomedical Engineering and Imaging Institute, Icahn School of Medicine at Mount Sinai, New York, NY, USA; ^3^ Invicro, London, UK; ^4^ Department of Applied Mathematics and Computer Science, Technical University of Denmark, Kongens Lyngby, Denmark; ^5^ Department of Mathematics, The University of Manchester, Manchester, UK; ^6^ Physikalisch-Technische Bundesanstalt, Braunschweig and Berlin, Germany; ^7^ School of Biomedical Engineering and Imaging Sciences, King’s College London, London, UK; ^8^ Institute of Nuclear Medicine, University College London, London, UK

**Keywords:** positron emission tomography, computed tomography, magnetic resonance imaging, electrical impedance tomography, tomography, imaging

## Abstract

This special issue focuses on synergistic tomographic image reconstruction in a range of contributions in multiple disciplines and various application areas. The topic of image reconstruction covers substantial inverse problems (Mathematics) which are tackled with various methods including statistical approaches (e.g. Bayesian methods, Monte Carlo) and computational approaches (e.g. machine learning, computational modelling, simulations). The issue is separated in two volumes. This volume focuses mainly on algorithms and methods. Some of the articles will demonstrate their utility on real-world challenges, either medical applications (e.g. cardiovascular diseases, proton therapy planning) or applications in material sciences (e.g. material decomposition and characterization). One of the desired outcomes of the special issue is to bring together different scientific communities which do not usually interact as they do not share the same platforms (such as journals and conferences).

This article is part of the theme issue ‘Synergistic tomographic image reconstruction: part 1’.

## 1. Introduction

Traditionally tomographic image reconstruction has focused on estimating two-dimensional or three-dimensional images from a single modality dataset and acquisition. In recent years, however, there have been significant developments in hardware and systems that allow extracting multi-parametric images from multiple datasets. Examples include multi-spectral X-ray Computed Tomography (CT), multi-sequence Magnetic Resonance Imaging (MRI), multi-modality imaging systems such as Positron Emission Tomography (PET) combined with MRI or CT, acquisitions from multiple time-points (i.e. time-lapse tomography) and imaging multiple radiotracers at the same or in separate sessions. In such cases, it is common practice to reconstruct several images independently, each corresponding to single or multiple parameters extracted from a single or multiple acquisitions. However, it is often advantageous, although challenging, to combine information to jointly reconstruct multiple images, while exploiting common features between these images. Such ‘synergistic image reconstruction’ methods are the topic of this special issue.

The special issue grew from the Symposium on Synergistic Image Reconstruction held in the autumn of 2019 in Chester (UK) which brought together many of the experts in the field (more information can be found in the corresponding website of the symposium: https://www.ccpsynerbi.ac.uk/symposium2019). The issue aims to create a collection of articles at the forefront of this subject from different research fields and application areas, including medical, biological and industrial imaging, to disseminate novel ideas, and present state-of-the-art research that will help accelerate new concepts and developments across all these different imaging fields.

The issue includes scientific articles across different application areas with one common theme: reconstruction of images by combining various different types of information. Several different approaches are used to solve the presented Inverse Problems. These include Bayesian methods, machine learning and deep learning algorithms as well as mathematical, physical, computational and when possible physiological modelling. The applications span from material sciences to scanning patients with advanced imaging such as for example integrated PET-MRI scanners. The special issue is separated in two different parts. The first issue (Part 1) includes three review articles and five research investigations. In both issues, there are articles which demonstrate the important utility of ideas when applied to answer application-specific questions. The imaging technologies featured in the first part of the special issue include but are not limited to spectral X-ray CT, MRI, PET-MRI, PET/CT, Electrical Impedance Tomography (EIT) and Quasi-Static Elasticity Imaging (QSEI). Many of these techniques have been developed only recently. For example, spectral CT has only recently appeared as a commercial clinical product and PET–MRI has been available for about 10 years as a combined clinical imaging machine, but there is still limited synergy between the two imaging techniques.

## 2. Contributions in this issue

The issue commences with a review by Arridge *et al.* [1] on synergistic image reconstruction methods which attempts to give the reader an update on the most recent published advances, while drawing similarities between the developments across different research fields.

MRI is a versatile imaging modality providing a range of different diagnostic information. Recently, quantitative MRI (qMRI), which produces maps of (bio)-physical parameters, has gained interest. These parameter maps are independent of any acquisition protocols or hardware issues and help reduce variability across different scans, devices, sites and patients leading to higher reproducibility. Clinically interesting parameters range from T1, T2 and T2* relaxation times to measuring blood flow velocities. The special issue continues with two reviews on qMRI.

Qi *et al.* give an overview of synergistic image reconstruction for multi-contrast cardiac parameter mapping. The data redundancy for mapping of multiple parameters can be used using e.g. sparsity or low-rank constraints during image reconstruction. This allows for higher undersampling factors (i.e. faster scans) while maintaining high image quality. One of the challenges is long reconstruction times. Deep learning-based parameter mapping is a promising alternative which ensures accurate parameter estimation in a clinically feasible time-frame [2].

The above review focuses on approaches where qMRI is carried out in two steps: in the first step, qualitative images are reconstructed from the acquired k-space data and in the second step, a model is used to obtain the quantitative parameters from these images. Model-based image reconstruction produces the parameters directly from the acquired k-space data without any intermediate image reconstruction. This leads to a non-linear reconstruction problem which is challenging to solve, but enables efficient qMRI. Wang *et al.* review model-based image reconstruction for different quantitative parameters and discuss several strategies to solve such problems [3].

**Figure 1.**
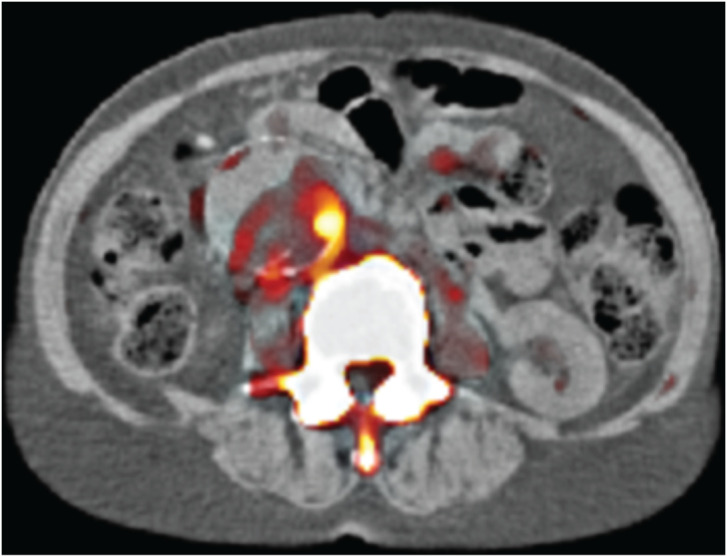
[^18^F]NaF PET image of a patient with abdominal aortic aneurysm as reconstructed with a synergistic algorithm using the CT image for guidance [7]. (Online version in colour.)

PET/CT scanning allows using CT images for guided PET reconstruction methods, where care needs to be taken with PET-only features. Deidda *et al.* demonstrate the usefulness of a synergistic image reconstruction method on a dataset of 72 human PET/CT scans primarily of patients with abdominal aortic aneurysms by using the kernelized expectation maximization (KEM) [4] and the hybrid KEM [5] algorithms. These two algorithms are compared with the conventionally used iterative image reconstruction algorithm: ordered subsets maximum likelihood expectation maximization (OSEM) [6]. The results illustrate that both KEM approaches can recover better the uptake of radioactivity in the aneurysms and potentially improve the diagnostic accuracy by up to 22% when compared to OSEM post-reconstructed filtered images [7] (figure 1).

Machine learning is becoming more and more important for medical image reconstruction. Lv *et al.* carry out a comparison of MRI image reconstruction approaches based on four different generative adversarial networks (GAN). The study was carried out on brain, knee and liver datasets which were retrospectively undersampled by factors of 2, 4 and 6. RefineGAN, which is the network achieving the overall highest image scores, showed high performance for all anatomies and all undersampling factors [8]. Future studies on multi-coil k-space data and including pathologies in the images are necessary.

Possible synergies exist not only between different types of reconstructed images. In simultaneous PET-MRI, both modalities are affected by the same physiological motion caused by breathing or the beating of the heart. Mayer *et al.* show that using images from both modalities for motion estimation through synergistic PET-MRI image registration allows for more accurate cardiac and respiratory motion estimation. This can improve the quality of [^18^F]NaF PET images of coronary plaques [9].

Within X-ray CT the development of new energy-discriminating detectors has motivated a tremendous research effort known as spectral CT with the possibility for improved material characterization and decomposition by synergistic exploitation of tomographic data measured over a range of X-ray energies. Perelli and Andersen propose a regularization method for multi-material decomposition in spectral CT [10]. This is based on denoising using a data-driven regularizer expressing complex prior structure. An efficient second-order optimization algorithm based on non-uniform random subsampling of the Hessian matrix is proposed to solve the problem. Experiments with synthetic and real datasets from a physical cylinder phantom demonstrate that the proposed method is capable of computing a reliable material decomposition producing comparable results with an existing method.

Hauptmann and Smyl present a synergistic approach for combining EIT and QSEI [11]. In EIT, an internal conductivity distribution is reconstructed from voltage measurements taken at the object boundary. In QSEI, the elastic modulus distribution is reconstructed from a displacement field measured within the object domain rather than at the boundary, and in this sense QSEI forms a complementary modality to EIT. Both EIT and QSEI have potential applications for example in medical imaging, however both are highly ill-posed, non-linear inverse problems. The authors propose a synergistic reconstruction method employing joint total variation to enforce structural similarity in particular common edges for the two modalities. Using simulation experiments for a case study of localization of cracks, they demonstrate improvements in image quality with the synergistic approach compared to single-modality TV-regularized reconstruction.

The contributions in this first part of the special issue provide an overview of the different scientific fields as well as important advances. We anticipate further exciting advances in Part 2, as well as several software-oriented papers. The latter describe open source software packages enabling researchers to explore synergistic image reconstruction methods. Taken together, we believe that this special issue highlights synergistic image reconstruction as a fertile area with scope for future research and ultimately fruitful application domains.

## Data accessibility

This article has no additional data.
